# Optimizing Nanosuspension Drug Release and Wound Healing Using a Design of Experiments Approach: Improving the Drug Delivery Potential of NDH-4338 for Treating Chemical Burns

**DOI:** 10.3390/pharmaceutics16040471

**Published:** 2024-03-27

**Authors:** Tomas L. Roldan, Shike Li, Christophe Guillon, Ned D. Heindel, Jeffrey D. Laskin, In Heon Lee, Dayuan Gao, Patrick J. Sinko

**Affiliations:** 1 Department of Pharmaceutics, Ernest Mario School of Pharmacy, Rutgers University, Piscataway, NJ 08854, USA; tlr86@scarletmail.rutgers.edu (T.L.R.); shikeli@pharmacy.rutgers.edu (S.L.); inheon@scarletmail.rutgers.edu (I.H.L.); dayuang@pharmacy.rutgers.edu (D.G.); 2 CounterACT Center of Excellence, Rutgers University, Piscataway, NJ 08854, USA; chg3@lehigh.edu (C.G.); ngs0@lehigh.edu (N.D.H.); jlaskin@eohsi.rutgers.edu (J.D.L.); 3 Department of Chemistry, Lehigh University, Bethlehem, PA 18015, USA; 4 Department of Environmental and Occupational Health and Justice, School of Public Health, Rutgers University, Piscataway, NJ 08854, USA

**Keywords:** design of experiments, nanosuspension, nitrogen mustard

## Abstract

NDH-4338 is a highly lipophilic prodrug comprising indomethacin and an acetylcholinesterase inhibitor. A design of experiments approach was used to synthesize, characterize, and evaluate the wound healing efficacy of optimized NDH-4338 nanosuspensions against nitrogen mustard-induced skin injury. Nanosuspensions were prepared by sonoprecipitation in the presence of a Vitamin E TPGS aqueous stabilizer solution. Critical processing parameters and material attributes were optimized to reduce particle size and determine the effect on dissolution rate and burn healing efficacy. The antisolvent/solvent ratio (A/S), dose concentration (DC), and drug/stabilizer ratio (D/S) were the critical sonoprecipitation factors that control particle size. These factors were subjected to a Box–Behnken design and response surface analysis, and model quality was assessed. Maximize desirability and simulation experiment optimization approaches were used to determine nanosuspension parameters with the smallest size and the lowest defect rate within the 10–50 nm specification limits. Optimized and unoptimized nanosuspensions were prepared and characterized. An established depilatory double-disc mouse model was used to evaluate the healing of nitrogen mustard-induced dermal injuries. Optimized nanosuspensions (A/S = 6.2, DC = 2% *w*/*v*, D/S = 2.8) achieved a particle size of 31.46 nm with a narrow size range (PDI = 0.110) and a reduced defect rate (42.2 to 6.1%). The optimized nanosuspensions were stable and re-dispersible, and they showed a ~45% increase in cumulative drug release and significant edema reduction in mice. Optimized NDH-4338 nanosuspensions were smaller with more uniform sizes that led to improved physical stability, faster dissolution, and enhanced burn healing efficacy compared to unoptimized nanosuspensions.

## 1. Introduction

Many drugs in development have poor aqueous solubility, which limits their bioavailability and effectiveness. An innovative approach to overcoming solubility challenges is to use nanosuspensions (NSs). NSs consist of nanometer-sized drug particles stabilized in a dispersion medium [1]. The reduced particle size of NSs increases the surface area of poorly water-soluble drugs, improving their solubility and dissolution rate [2]. The small size of NS particles can also allow them to pass through biological barriers to reach target tissues or cells, and they can be engineered to release drugs in a controlled manner, enabling sustained delivery [3]. The formulation and production of NSs can be complex, requiring careful optimization of various parameters to ensure their quality and stability [4]. Top-down NS fabrication methods involve reducing the size of larger particles to the nanoscale through techniques like milling, while bottom-up approaches assemble NSs from molecular components through processes such as antisolvent precipitation or chemical synthesis. One popular bottom-up method is sonoprecipitation.

Sonoprecipitation has gained prominence in formulating poorly water-soluble drugs, as ultrasonication enhances their dissolution rates by reducing particle sizes [5,6]. This process uses cavitation, which leverages ultrasonic energy to break apart larger particles by creating microscopic bubbles that collapse and release energy to facilitate particle fragmentation. The principle underlying sonoprecipitation is antisolvent precipitation, where a solvent containing the drug is introduced into an antisolvent where the drug has limited solubility. New solid particles are created as the solvent diffuses, drug supersaturation occurs, and nucleation begins.

The outcome of sonoprecipitation is influenced by various parameters. The antisolvent/solvent ratio (A/S) primarily dictates the degree of supersaturation. A higher A/S provides more energy to initiate and maintain the nucleation process. Dose concentration (DC) refers to the concentration of the active pharmaceutical ingredient (API) within the NS, expressed as a percentage of weight of the drug relative to the volume of the suspension, which contributes to supersaturation. The drug/stabilizer ratio (D/S) in NS synthesis affects the steric hindrance on the surface of the nanoparticles, which is essential for preventing particle aggregation and ensuring the physical stability of the NS. Sonication amplitude and time determine the intensity and duration of the cavitation forces, with high amplitudes and longer sonication times resulting in smaller and more uniform nanoparticles.

Design of experiments (DOE) is a powerful statistical tool used in research to determine the effect of different variables (factors) on a particular outcome [7]. By carefully planning experiments and choosing factor levels that adequately cover the design space, DOE lets researchers obtain the maximum amount of information with the fewest experimental runs, thus saving time and resources. DOE can help identify ideal drug formulation and manufacturing conditions, such as the concentration, pH, and temperature influencing drug solubility, stability, and bioavailability [8]. Additionally, DOE can help identify and reduce the impact of potential sources of variability that can affect product quality and stability, including process variability, formulation variability, and raw material variability [9,10,11,12]. The DOE methodology uses several core elements that help with systematic process understanding, thus enabling optimization and quality control. Critical Process Parameters (CPPs) are essential operational variables within a manufacturing system that influence the quality of the final output. These variables interface with Critical Material Attributes (CMAs), which embody the physical, chemical, or biological properties of the raw materials used in production [13]. The interaction of CPPs and CMAs informs the Critical Quality Attributes (CQAs), quantifiable properties that delineate the desired product quality thresholds. The Box–Behnken design (BBD) is a response surface methodology (RSM) design used in the antisolvent precipitation NS DOE literature [14,15,16]. A significant advantage of BBD is its ability to generate higher-order response surfaces with fewer required runs than traditional factorial techniques like full factorial, fractional factorial, and central composite designs [17]. This run requirement efficiency allows for a more thorough exploration of the response surface with reduced experimental effort and fewer required resources. The BBD center points used in RSM enable replication, thus helping estimate pure error and tests for lack of fit and detecting curvature in the relationship between the response and the factors.

Bi-functional alkylator vesicants like sulfur mustard (SM) and mechlorethamine hydrochloride, also known as nitrogen mustard (NM), injure the eye, skin, and lung tissues [18]. Both SM and NM skin exposure cause a severe inflammatory response, including dermal edema [19]. Using edema as an outcome measure for wound healing is a straightforward and time-efficient means of determining vesicant-induced inflammatory response. Treatment of NM burns requires prompt and effective medical interventions (i.e., countermeasures). NDH-4338, a prodrug that shows activity against NM and SM burns, comprises indomethacin, a non-steroidal anti-inflammatory drug, and 3,3-dimethyl-1-butanol, an acetylcholinesterase inhibitor [20]. NDH-4338 is highly lipophilic (ClogP 7.87) and requires solubility enhancement for acceptable therapeutic efficacy [21].

Topical formulations used to treat SM skin burns include solvent mixture, cream, ointment, gel, and hydrogel vehicles [20,22,23,24,25]. For NM skin burn treatments, vehicles are comprised of organic and polymeric solvents, ointments, creams, and hydrogels [23,26,27,28,29,30,31,32,33]. Chang et al. reduced SM-induced edema by administering NDH-4338 in a dimethyl isosorbide/ethyl oleate vehicle [20]. Subsequently, NDH-4338 was utilized in a lanolin/PEG400 formulation by Joseph et al. to inhibit SM-induced mast cell degranulation [25]. While initial NDH-4338 formulations produced by our team were effective in treating NM burns in mice, the onset of action may have been delayed due to slow release from the viscous lanolin/PEG400 vehicle, as more viscous formulations have been shown to slow dissolution and reduce dermal drug penetration [28,33,34]. Additionally, the NDH-4338 formulations had to be applied to NM skin burns four times daily (QID) to achieve the observed efficacy. Since viscous vehicles require more force to distribute them across the skin, this could contribute to discomfort or pain during the application process, particularly when frequently applied. Thus, less frequent applications with a less viscous formulation could enhance dermal bioavailability and improve comfort during application.

The current study focuses on designing and evaluating an NS formulation at a twice-a-day (BID) dosing regimen. Vitamin E TPGS was chosen as a stabilizer for this study due to its high biocompatibility, as well as its amphiphilic nature that improves drug solubility and enhances drug permeation [35]. Using DOE, the stability, morphology, dissolution rate, and skin burn healing efficacy of an optimized NDH-4338 NS were compared to an unoptimized NS formulation.

## 2. Materials and Methods

### 2.1. Reagents

NDH-4338 was synthesized as previously described [36]. Vitamin E TPGS NF Grade (Vitamin E TPGS) was from Eastman Chemical Company (Kingsport, TN, USA) and was used as the NS stabilizer. Mechlorethamine hydrochloride (NM) was purchased from Sigma-Aldrich (St. Louis, MO, USA). Ultrapure water (Milli-Q^®^ Advantage A10^®^, Type 1 water, MilliporeSigma, Burlington, MA, USA, water) was used throughout the study. Isoflurane was obtained from Covetrus North America (Portland, ME, USA).

### 2.2. Preparation of NDH-4338 Nanosuspensions

NDH-4338 NSs were prepared using sonoprecipitation, an evaporative-ultrasonication method [37]. Briefly, NDH 4338 was dissolved in acetone (i.e., the solvent) and injected all at once using a needle (30 G ½) into water (i.e., the antisolvent) containing Vitamin E TPGS, and the resulting mixture was placed in an ice bath. The ultrasonic probe was placed into the antisolvent solution and operated with one minute off between each minute using a Branson Sonifier 450 (Branson Ultrasonics, Danbury, CT, USA) at amplitude settings of 50, 60, and 70% and processing times of 5, 10, and 15 min. During the pause, the probe was removed from the mixture to let it cool. All NSs were lyophilized (FreeZone Plus 4.5, Labconco, Kansas City, MO, USA) with liquid nitrogen for 72 h with vacuum pressure below 20 mTorr and condenser temperature at −80 °C. Samples were reconstituted with refrigerated (2–8 °C) water and gently vortexed.

### 2.3. Preliminary Screening on Factors Involved in Evaporative-Ultrasonication

**Figure 1:**
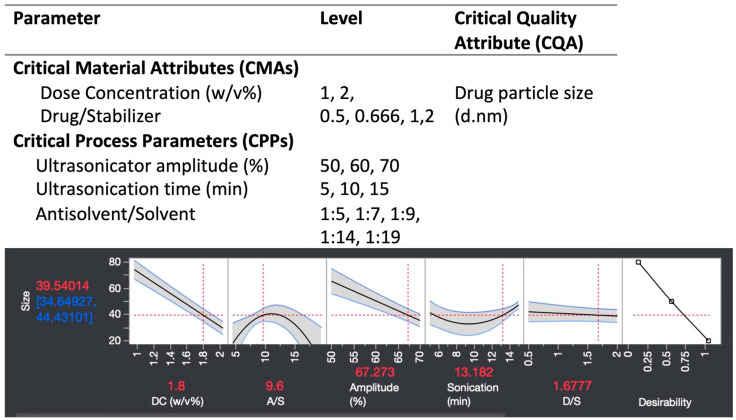
A preliminary screening of the effects of CMAs and CPPs on the particle size. Preliminary experiments explored operative ranges of five factors (table). A response surface analysis on the preliminary results revealed that higher ultrasonicator amplitude and dosing concentration (DC) led to lower particle size. The antisolvent/solvent ratio (A/S) displayed a biphasic effect. An increased drug/stabilizer ratio (D/S) corresponded to a smaller particle size. Therefore, the ratio was further increased beyond the tested range for the DOE. The 70% ultrasonicator amplitude and 10 min processing time were fixed as these settings yielded the smallest particle sizes, chosen for the ease of configuring the analog ultrasonicator and to save experimental time. Three variables, A/S, DC, and D/S were further subjected to a design of experiment.

Five factors involved in the NS fabrication process, including the CMA and CPP, were first identified (Figure 1). Reducing NS particle size was integral for maximizing NDH-4338 solubility enhancement and was thus defined as the CQA. An initial evaluation was performed to determine the range for each factor. Fifteen experiments were conducted to screen DC and D/S as CMA; A/S, ultrasonicator amplitude, and processing time as CPP; and particle size as the CQA. The range of experiments tested a DC of 1 and 2% *w*/*v*; a D/S of 0.5, 0.666, 1, and 2; and the previously described ultrasonicator amplitude settings and processing times, as shown in the table of Figure 1. Particle size was measured using dynamic light scattering (DLS) (see below). NS size results were analyzed using the RSM to evaluate and visualize the effects of the CMAs and CPPs on the CQA using JMP Pro 16 (JMP, Cary, NC, USA).

### 2.4. Design of Experiments

**Figure 2:**
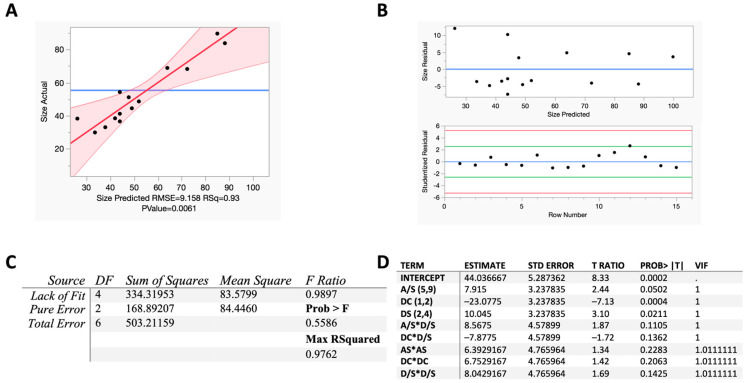
Model diagnostics confirm that the DOE model is robust. The actual by predicted plot, measuring particle size in nm, shows that the model fits the data well and accounts for most of the observed variation (**A**). All points fall close to the regression line, and the R-squared is 0.93, meaning 93% of the data variation in the response is explained by the model. The shaded red regions depict a 95% confidence interval. The plot has a *p*-value less than 0.05, meets validation criteria, and has a root mean squared error (RMSE) of 9.158, which is not outside of a normal value as it scales with response data. Residual by predicted plot and studentized residuals show that the residual is constant, and there is no indication of potential outliers (**B**). The null hypothesis that no variable related to a response variable is rejected according to the *p*-value for the goodness-of-fit test (**C**). Prob > F is greater than 0.05, indicating that there is no lack of fit. Parameter estimates show that a variable inflation factor (VIF) is less than 5, indicating no multicollinearity, and each factor can be included in the model (**D**).

As informed by the factor screening analysis, three factors, A/S, D/S, and DC, were subjected to DOE for further systematic exploration. Fifteen NS formulations were then prepared using a BBD and synthesized according to the factor settings outlined in Figure 2, and their sizes and PDIs were determined. The NS formulations’ sizing data were then fit using standard least squares regression to generate the response surface model shown below:

Y = m_0_ + m_1_X_1_ + m_2_X_2_ + m_3_X_3_ + m_11_X_1_^2^ + m_22_X_2_^2^ + m_33_X_3_^2^ + m_13_X_1_X_3_ + m_23_X_2_X_3_ + m_12_X_1_X_3_

where Y is the CQA response variable, i.e., NS size; X_1_, X_2_, and X_3_ are the explanatory variables, i.e., the three factors used to construct the BBD; and the m_0_, m_1_, m_2_, and m_3_ are the model coefficients representing the primary (m_1_, m_2_, m_3_), quadratic (m_11_, m_22_, m_33_), and interaction (m_12_, m_13_, m_23_) effects.

Diagnostic assessments are analytical techniques used to evaluate a statistical model’s assumptions, performance, and reliability [38]. To assess the quality of the obtained model, diagnostic tests such as R-squared in the actual by predicted plot, lack of fit, multicollinearity determined by a variable inflation factor (VIF), residual by predicted plot, and studentized residuals were evaluated with JMP Pro 16.

Two optimization approaches were used to identify the most robust factor settings for producing the smallest particle size and the lowest defect rate. The first approach, “maximize desirability”, utilizes the acquired response surface model to explore multidimensional factors and responses, aiming to identify the optimal settings for achieving the minimum particle size using the Desirability Profiler functions in JMP Pro 16. The maximize desirability function generates optimal factor settings across multiple responses based on predefined targets, with “desirability” quantifying how closely these targets are achieved [39,40,41].

For the second approach, the “simulation experiment” introduces randomness into the design space to test many conditions to ensure the most robust settings [42]. This experiment comprised 128 Monte Carlo simulations and explored the full factor range to predict the defect rate from the target specification limits. NS optimization specification limits were set at 10–50 nm, encompassing the 40 nm particle size indicated by Mahe et al., to facilitate dermal delivery by penetrating mouse hair follicles [43]. While the range brackets the 40 nm size, it is weighted towards smaller particles to prioritize enhancing dissolution. The simulation experiment results were analyzed using a Gaussian process script to find the optimal factor settings that minimize the defect rate.

The optimization strategy that resulted in the smallest predicted size, balanced with the lowest defect rate, was selected for the optimized NS factor settings. The formulation resulting from these finalized parameters was called the “optimized NS”. Before the DOE was conducted, the starting parameters were an A/S = 10, DC = 2% *w*/*v*, D/S = 2, amplitude = 5/10, and ultrasonication time = 6 min. The resulting “unoptimized NS” had a size of 107.7 nm and a polydispersity index (PDI) of 0.250 (Figure S1). The optimized NS was then synthesized and subjected to a test equivalence distribution analysis to validate the predictive model. A 95% confidence interval was used to determine if the particle size was equivalent to the predicted value.

### 2.5. Characterization of NDH-4338 Nanosuspensions

#### 2.5.1. Particle Size and Polydispersity Index (PDI)

Particle size and PDI of the NSs were measured using a Zetasizer Nano ZS90 (Malvern Instruments, Malvern, UK) by DLS technique at 15 °C. The studies were conducted in triplicate.

#### 2.5.2. Particle Morphology

Transmission electron microscopy (TEM) was used to capture images of NS particles from optimized and unoptimized formulations at magnifications ranging from 10,000× to 160,000×. Samples were dispensed onto a 400 mesh Formvar/Carbon-coated copper grid and stained with a 1% uranyl acetate solution. The grid was carefully positioned into a sample holder using N5 Dumoxel tweezers (Dumont, Montignez, Switzerland) and inserted into the TEM for imaging.

#### 2.5.3. Dissolution Studies and Modeling of NDH-4338 Nanosuspensions

##### In Vitro Dissolution Studies

Bulk NDH-4338 powder, as well as freeze-dried optimized and unoptimized NDH-4338 NSs, were added to 100 mL of a preheated 50% MeCN/50% water solution, maintained at 32.5 °C and agitated at 200 RPM. Sink conditions were carefully maintained throughout the experiment. At designated time intervals (5, 10, 25, 50, 100, 130, 180, and 240 min), receptor fluid samples were withdrawn and centrifuged, and the resulting supernatant was analyzed using HPLC/UV for NDH-4338 quantification.

##### Dissolution Data Modeling

Dissolution data were analyzed using DDSolver, a Microsoft Office Excel 2007 plug-in, to determine the mathematical model that best described the observations [44]. The Akaike Information Criterion (AIC) is a statistical measure used for model selection assessing the trade-off between goodness of fit and complexity [45]. A lower AIC value indicates a better fit when comparing models with varying numbers of parameters. The Model Selection Criteria (MSC) is a modified reciprocal form of the AIC and has been normalized to be independent of data point scaling [44]. Preferable models had larger MSC and R-squared values and smaller AIC values.

Ten mathematical models, including Higuchi, Higuchi F0, Zero Order, Zero Order F0, First Order, Korsmeyer–Peppas, and Weibull models, were applied using DDSolver to analyze the dissolution profiles of NDH-4338 in bulk powder, optimized, and unoptimized NS. The formulas for each model utilized by DDsolver specifically were detailed by Zhang et al. [44]. Of these models, the three with the most favorable R-Squared, MSC, and AIC values (Higuchi F0, Korsmeyer–Peppas, and Weibull 4) were compared in-depth to select the most robust model. The Weibull 4 model’s parameters are α (scale), β (shape), Ti (lag time), and Fmax (maximum fraction of drug release at infinite time). Higuchi F0 features the Higuchi release rate constant (kH), while the Korsmeyer–Peppas model includes kKP and *n* variables, representing the Korsmeyer–Peppas release rate constant and diffusional exponents, respectively.

#### 2.5.4. Re-Dispersibility and Stability

The NS was freeze-dried and then reconstituted in water to 2% *w*/*v* to assess short-term stability and re-dispersibility. Particle size and PDI were measured using dynamic light scattering (DLS) immediately after reconstitution. The reconstituted NS was then stored at 2–8 °C. Particle size and PDI were measured repeatedly at 3 subsequent time points: 24, 72, and 96 h.

Although zeta potential is commonly utilized in nanomedicine to assess stability based on charge repulsion mechanisms, it mainly applies to electrostatically stabilized systems [46]. The optimized NS formulation used Vitamin E TPGS as a steric stabilizer, which mitigates NS particle aggregation primarily through steric hindrance rather than electrostatic repulsion. Therefore, zeta potential measurement was not performed, as the stability was maintained through physical means across the 96 h period, as evidenced by consistent particle size and PDI values less than 0.3 (indicating a homogenous size distribution) [47].

#### 2.5.5. High-Performance Liquid Chromatography (HPLC) Quantitation

Quantitative analysis was conducted using the e2695 Alliance System (Waters, Milford, MA, USA), an HPLC system equipped with a photodiode array detector capable of ultraviolet (UV) and visible absorbance between 200 and 600 nm. The mobile phase for HPLC was composed of (A) water with 0.05% (*v*/*v*) trifluoroacetic acid (TFA) and (B) MeCN with 0.05% TFA (*v*/*v*). An injection volume of 10 mL was used, and chromatographic separations were performed on a Waters Symmetry C18 column (Waters, Milford, MA, USA). A standard curve of NDH-4338 was prepared in the concentration range 1–200 μg/mL, exhibiting a linear coefficient of determination (R^2^) of 0.994. The HPLC run time was 10 min, with NDH-4338 demonstrating an analyte retention time of 7 min at a detection wavelength of 212 nm. Validation of the analytical procedure included testing for specificity, selectivity, linearity, precision, and accuracy. For encapsulation efficiency (EE) assessment, vortexed NS samples were diluted 180 times with MeCN and subjected to HPLC/UV quantitation. The assay sample concentrations fell within the quantifiable linear range.

### 2.6. Effects of NDH-4338 on NM-Induced Edema

Female CD-1 mice (8–9 weeks old) were procured from Charles River Laboratories (Wilmington, MA, USA). Upon arrival, mice were acclimated for at least one week before the studies began. The mice were housed in a university animal facility under a 12 h light/dark cycle and maintained at a controlled temperature, with ad libitum access to food and water. To test the wound healing efficacy of the optimized (OT) and unoptimized (UT) treatment formulations, the depilatory double disc mouse (DDD) model was utilized as previously described [33].

The experimental groups consisted of naïve control, NM burn control (NM), and optimized and unoptimized NS treatment groups (OT and UT) at a dose of 0.4 mg NDH-4338 equivalent per vesicant treated area, along with their respective vehicle control groups (OV and UV). These vehicles were made with the same material attributes and processing factors as the respective optimized (OT) and unoptimized (UT) NS formulations.

A power analysis was performed using G*Power 3.1.9.6 to determine the appropriate group size for the animal studies. Using a two-tailed *t*-test with an alpha level of 0.05 and a desired power of 0.8, means and standard deviations derived from a pilot study were utilized. The optimal group size was determined to be 7 per group, ensuring robustness and reliability in the experimental design.

Our previously established DDD NM skin burn model was used for the mouse studies [33]. In brief, NM (5 μmol) was applied to two filter discs surmounting the dorsal lumbar region of mice for 6 min. One hour after NM administration, 0.4 mg NDH-4338 equivalent in 20 µL of formulation was applied to the vesicant-exposed regions for each NS. Similarly, 20 µL of the respective vehicles were applied to these regions for the vehicle control groups. Throughout the treatment, mice were anesthetized with isoflurane for 10 min before being monitored until wakefulness resumed. The treatments were applied twice (every 12 h), after which the mice were euthanized using CO_2_. Skin punch (12 mm, Acuderm Inc., Ft. Lauderdale, FL, USA) biopsies were then obtained from the treated areas and weighed with an analytical balance. Edema was assessed by subtracting the average punch biopsy weight of the naïve control group from each treated group to determine skin weight gain.

### 2.7. Statistical Analysis

The DOE analysis, including BBD, model fitting, model diagnostics, and validation, was conducted using JMP Pro 16 (JMP, Cary, NC, USA). Statistical analyses were performed using Prism 6 and 10 (GraphPad Software, San Diego, CA, USA). Statistical significance was determined using one-way ANOVA analysis with Tukey’s multiple comparison test, with *p* < 0.05 considered significant. Results are reported as mean ± standard deviation (SD). G*Power version 3.1.9.6 was used to conduct a power analysis to determine the appropriate group size for the in vivo burn healing efficacy study.

## 3. Results and Discussion

### 3.1. Factor Screening Analysis

Preliminary screening experiments and analysis to determine appropriate factors and ranges to be included in a designed experiment is a crucial scoping step in the DOE workflow. The screening also helps balance experiment size with generating impactful results. The five CMA and CPP factors and ranges tested are shown in Figure 1. The response surface analysis indicated DC and sonication amplitude had the most dominant linear effect on decreasing particle size over the range studied, with higher DC and amplitude associated with smaller particle sizes (Figure 1). The effects of the A/S ratio and sonication time on particle size were more complex and displayed a biphasic curvature. The particle size distribution manifested a peak and valley in the tested A/S and sonication time range. This implied that potential optima for reducing particle size exist for A/S ratio and sonication time, beyond which further increasing these factors had diminishing returns or a counterproductive effect. D/S showed a flat yet negative correlation with particle size (Figure 1).

The factors and ranges for the BBD were set based on the screening analysis. Ultrasonication processing factors were fixed to 70% amplitude and 10 min, as the smallest particle sizes were observed at these approximate settings. Additionally, these two parameters were chosen for the ease of accurately configuring the analog ultrasonicator and to optimize experimental efficiency. The A/S ratio was set from 5 to 9 for the BBD, as this range had a steeper slope than 11 to 15 (Figure 1). Increasing the D/S ratio resulted in smaller particle sizes; thus, the range was increased from 2 to 4 (Figure 1). Although DC greater than 2% NDH-4338 may lead to smaller particle size, it was capped at 2% to balance efficacy and potential lethality of treatment to the mice: (1) efficacy was established at a 1% (approximately a 0.2 mg dose) concentration, and (2) lethality in mice was observed at 3% (approximately a 0.6 mg dose) concentration and higher doses [28]. Three variables, A/S, DC, and D/S, were further subjected to the BBD analysis.

### 3.2. BBD Model Fitting and Model Diagnostics

A BBD analysis involving three factors and three levels was conducted following the preliminary study analysis. The DOE was segmented into three sections: CMAs, CPPs, and CQAs. CMAs were DC and D/S ratio, the CPP was A/S ratio, and the CQA was NS particle size. The A/S range was set from 5 to 9, 1 to 2 for the DC, and 2 to 4 for the D/S, as respective low- and high-value independent process variables within a continuous modeling type. These coded levels were used to generate a 15-run BBD with JMP Pro 16 (Table 1). For the BBD data in this study, the particle size ranged from 30 to 104 nm, the PDI ranged from 0.002 to 0.365, and the EE ranged from 78.1 to 99.7%. Gajera et al. used a BBD to generate a clotrimazole NS by sonoprecipitation and achieved particle sizes ranging from 92.30 to 560.3 nm, with the optimal formulation comprising 131.7 nm [14]. Kuk et al. also used a BBD for resveratrol NSs prepared using an antisolvent precipitation technique that ranged from 33.7 to 165.5 nm, with the optimized formulation being 46.3 nm [15]. The results presented here generated particle sizes ranging from 29.92 to 103.5 nm and an optimized NS size of 29.74 nm with a PDI of 0.080 (Figure S1). Vitamin E TPGS has been established in drug delivery as a prodrug carrier and is used for its ability to form micelles and nanoparticles [48]. Ghosh et al. used Vitamin E TPGS as an NS stabilizer in conjunction with HPMC [49,50]. The present study is the first to employ Vitamin E TPGS as a surfactant stabilizer for NSs with no other excipients, presenting a promising option for delivering other poorly water-soluble compounds.

Three critical factors were selected from the initial evaluation data: antisolvent/solvent ratio (A/S), dose concentration (DC), and drug/stabilizer ratio (D/S). These selected levels for A/S, DC, and D/S for the DOE are based on observed trends from the factor screening analysis. A 15-run Box–Behnken Design was constructed using JMP Pro 16 to minimize particle size. The independent process variables and their coded levels were A/S (Low Value: 5, High Value: 9), DC (Low Value: 1, High Value: 2), and D/S (Low Value: 2, High Value: 4). “Pattern” refers to a specific arrangement of experimental runs designed to explore the effects of variables on an outcome efficiently. The symbols “0”, “+”, and “−” in the design matrix represent the coded levels of the independent variables: “0” denotes the center point (or baseline level) of the variable, “+” indicates a higher level, and “−” indicates a lower level. The 000 pattern represents the three center points of the Box–Behnken Design.

The statistical significance of each term in the model fit is shown in Table S1. The exclusion of the insignificant interaction term AS*DC (*p* = 0.45193) decreased *p*-values for other model terms, enhancing the model’s overall significance.

For model diagnostics, the actual by predicted plot demonstrated an R-squared of 0.93 with a *p*-value of 0.0061, meeting statistical verification criteria (Figure 2A) [51]. The plot has a root mean squared error (RMSE) of 9.158, which is not outside of a normal value as it scales with response data [52]. The model exhibited no lack of fit (Prob > F = 0.5586) and adequately fitted the data, with two degrees of freedom for pure error, four degrees for lack of fit, and six for total error (Figure 2B) [53]. There was no indication of potential outliers, and variance inflation factor (VIF) estimates of less than five confirmed no multicollinearity, indicating that each parameter could be included in the model (Figure 2C,D) [54]. Fulfilling these diagnostic measures indicates that the model sufficiently describes the experimental data using the modeling technique. The model diagnoses reveal no serious deficiencies or deviations. The achieved model is expressed as below:

y = 44.036667 + 7.915 × (A/S) − 23.0775 × DC + 10.045 × (D/S) + 8.5675 × (A/S × D/S) − 7.8775 × (DC × D/S) + 6.3929167 × (A/S)^2^ + 6.7529167 × DC^2^ + 8.0429167 × (D/S)^2^

### 3.3. Factor Setting Optimization and Validation

**Figure 3:**
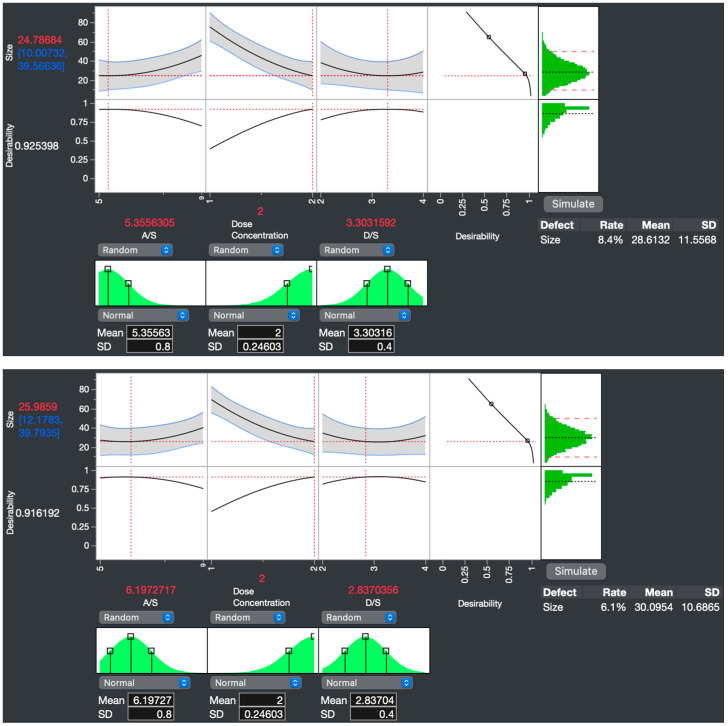
Simulation experiment optimization shows the factor settings to produce the smallest nanosuspension (NS) with the lowest defect rate. The maximize desirability optimization approach (top) was used to optimize the response variable, thereby reducing the particle size as a priority. The simulation experiment approach (bottom) specialized in decreasing the defect rate from the 10–50 nm specification limits. Maximize desirability and simulation experiment optimization approach decreased the defect rate from the initial 42.2% to 8.4 and 6.1%, respectively. For the simulation experiment, the defect rate was 2.3% lower than the maximize desirability approach, with a narrower CI range and a minor trade-off in predicted size. Therefore, to ensure maximal robustness in reducing NS particle size, the factor settings for the optimized NS were set to those of the simulation experiment: antisolvent/solvent ratio (A/S) of 6.2, dose concentration (DC) of 2, and drug/stabilizer ratio (D/S) of 2.8.

After maximizing the desirability function, the defect rate for the 10–50 nm specification limits decreased from 42.2 to 8.4%, with a predicted size of 24.78684 nm [CI range: 10.00732–39.56636] (Figure 3, top). The factor settings for this first optimization approach were DC of 2% *w*/*v*, A/S of 5.4, and D/S of 3.3. The second optimization approach aimed to minimize the defect rate further. A Gaussian process script of the simulation experiment showed factor conditions that lowered the defect rate to 6.1%, with a predicted size of 25.9859 nm [CI range: 12.1783, 39.7935]. The predicted NS particle sizes from the two approaches were very similar.

**Figure 4:**
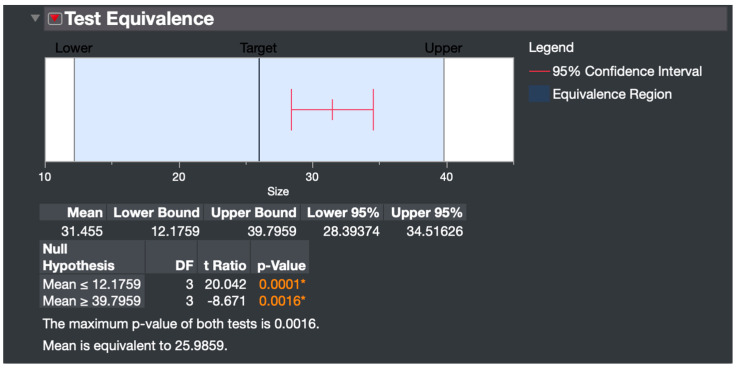
A Test equivalence distribution analysis validates the model performance. The particle sizes of four formulations prepared at the optimal conditions were statistically equivalent to the predicted value. *, statistical significance at *p* < 0.05.

The factor settings from the minimizing defect rate optimization approach were therefore chosen over those of the minimizing particle size approach, as the defect rate was 2.3% lower with a corresponding narrower CI range, with a minor trade-off in predicted size of only 1.19906 nm (Figure 3, bottom). Thus, the factor conditions of the optimized formulation were DC of 2% *w*/*v*, A/S of 6.2, and D/S of 2.8. NSs were fabricated using these predicted factor settings, and particle size was measured. The test equivalence distribution analysis showed the mean of four samples prepared at the optimal conditions was equivalent to the predicted size of 25.9859 nm, validating the predictive capability of the model (Figure 4). In equivalence testing, the “Difference Considered Practically Zero” is the threshold below which the true mean is practically equivalent to the hypothesized mean [55]. In this study, the difference was set at 13.8076, calculated as half the confidence interval range around the hypothesized mean of 25.9859 nm at a 95% confidence level.

### 3.4. Re-Dispersibility and Stability

**Figure 5:**
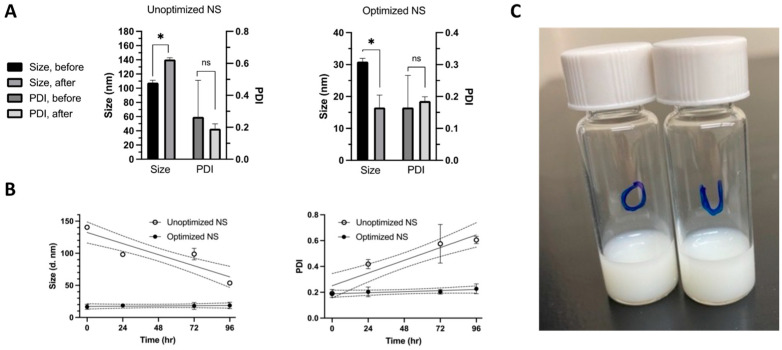
The optimized nanosuspension (NS) exhibited superior particle size and polydispersity (PDI) stability. The size and PDI of the optimized NS were determined by dynamic light scattering before and after reconstituting the lyophilized powder, as well as at 24, 72, and 96 h time points. After reconstitution, the size of the optimized NS remained smaller than before, and the PDI stayed under the quality data threshold of 0.3 for the entire stability test (**A**). The optimized NS also demonstrated a more stable particle size and PDI than the unoptimized NS (**B**). Each column or point represents the mean value ± SD (*n* = 3). Linear regression was applied to the observed data, and the area between the dotted lines represents the 95% CI. *, *p* < 0.05; ns, *p* > 0.05 by two-tailed Student’s *t*-test of before vs. after lyophilization and reconstitution. A representative macroscopic image shows that the optimized (O) and unoptimized (U) NSs are white and have a similar appearance (**C**).

For the optimized NS, the particle size after synthesis of 30.96 nm was reduced to 16.58 nm upon reconstitution (Figure 5A). This reduction in NS particle size after freeze-drying may be attributed to reverse Ostwald ripening. In this process, smaller particles become more stable and form at the expense of larger ones [56]. This phenomenon can be caused by the increased concentration of Vitamin E TPGS in the reduced reconstitution volume, promoting a uniform and dense protective layer around each particle. The optimized NS displayed adequate stability throughout the 96 h time course, with sizes ranging from 16.58 nm to 18.69 nm and PDIs ranging from 0.186 to 0.227, indicating a narrow size distribution with modest changes (Figure 5B) [47]. In contrast, the unoptimized NS was 107.9 nm after synthesis and was reconstituted at 140.5 nm (Figure 5A). The size varied significantly throughout the 96 h time course, ranging from 98.25 nm to 53.71 nm, with PDIs ranging from 0.418 to 0.604, indicating a highly polydisperse sample (Figure 5B). The negative slope for the unoptimized size reflects a decrease in particle size over the 96 h period, while a positive PDI slope reveals an increase in heterogeneity (Table S2). The flat lines (~0 slope) demonstrate that size and PDI remained consistent throughout the study, confirming the optimized NSs were stable. A representative macroscopic image shows that the NS formulations are white and appear similar (Figure 5C).

### 3.5. NS Morphology by TEM Imaging

**Figure 6:**
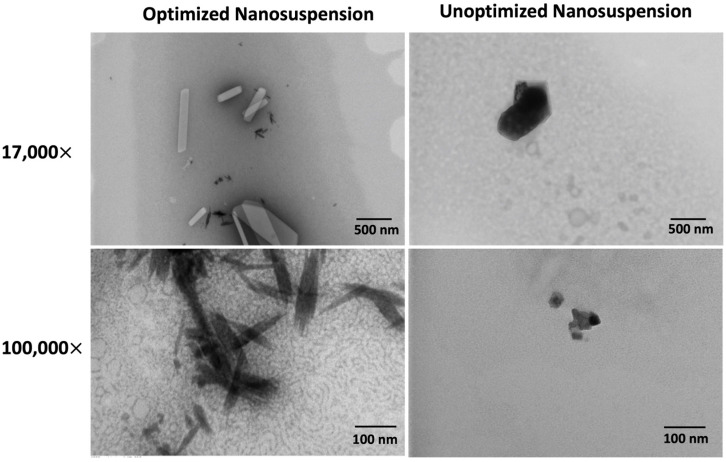
Transmission electron microscopy (TEM) images reveal that the optimized nanosuspension (left) possesses a more crystalline nanocrystal than unoptimized amorphous nanosuspensions (right). TEM images of the optimized and unoptimized nanosuspension formulations at 17,000× (top) and 100,000× (bottom) magnifications. Optimized nanocrystals show size-dependent morphological and color changes, transitioning from dark, slender, shard-like structures at ~30 nm to white, well-defined, dynamically shaded structures in the micron range. Unoptimized nanocrystals predominantly exhibit dark, amorphous, blob-like morphologies, with slight indications of more crystalline structures in larger particles.

Distinct morphological and color differences were observed between the optimized and unoptimized NSs. A noticeable color shift from black to white was observed in the optimized NS, which might indicate size-dependent alterations in light scattering and diffraction properties [57]. The optimized NS contained nanocrystals exhibiting a range of morphologies, with the smallest particles appearing as slender, dark, shard-like structures bearing striations indicative of a crystalline structure. (Figure 6) Larger crystals within the same formulation appeared white, bearing sharply defined edges and dynamic shading (Figure 6). The unoptimized NS predominantly comprised amorphous particles, appearing as dark, blob-like structures. Some larger particles indicated a more crystalline morphology, yet the general trend was toward amorphousness. Notably, no white-colored particles were observed within the unoptimized NS (Figure 6). The crystalline nature of the optimized NS could explain the improved physical stability compared to the amorphous unoptimized formulation, as the increased lattice energy in crystalline nanomedicines allows for greater stability compared to their amorphous counterparts [58].

TEM was initially used as an orthogonal sizing method for DLS. However, discrepancies in particle sizes were observed between the two techniques. While DLS measurements account for the hydrodynamic layer of wet samples, TEM measurements are performed on dried samples, introducing the potential for aggregation during the drying process [59,60]. DLS assumes a spherical shape for particles during size calculation [61]. In the case of non-spherical or irregularly shaped particles, such as the shard-like and amorphous particles observed in the optimized and unoptimized NSs, DLS can potentially misestimate the actual size due to the influence of shape and orientation [61,62].

### 3.6. In Vitro Dissolution

The Higuchi F0 model demonstrated good fits with R-squared values from 0.814 to 0.972 (Table 2). AIC values were between −27.35 and −45.92, and MSC values spanned 1.513 to 3.099. The Korsmeyer–Peppas model demonstrated higher R-squared values than that of Higuchi F0, ranging from 0.938 to 0.975 (Table 2). Corresponding AIC values were lower than those of Higuchi F0, spanning from −33.38 to −48.40, and the MSC values were higher, ranging from 1.823 to 3.211.

The Weibull 4 model was selected for dissolution modeling, as the highest R-squared values, lowest AIC values, and highest MSC values were observed (Table 2). R-squared values ranged from 0.973 to 0.989, AIC values from −40.72 to −57.04, and MSC values 2.903 to 3.676. Fmax values for the optimized NS were above 1, indicating a quicker complete dissolution than the unoptimized NS at 0.990, whereas the bulk powder only achieved about 50% dissolution with an Fmax value of 0.541 (Table S3). The β values less than 1 for the optimized NS suggest that the dissolution rate increases over time, while it decreases for the bulk and unoptimized NS (1.792 and 1.111, respectively). Lastly, the lower α (scale) parameter values for the optimized NS suggest a quicker dissolution rate relative to the unoptimized NS and bulk powder (Table S3).

**Figure 7:**
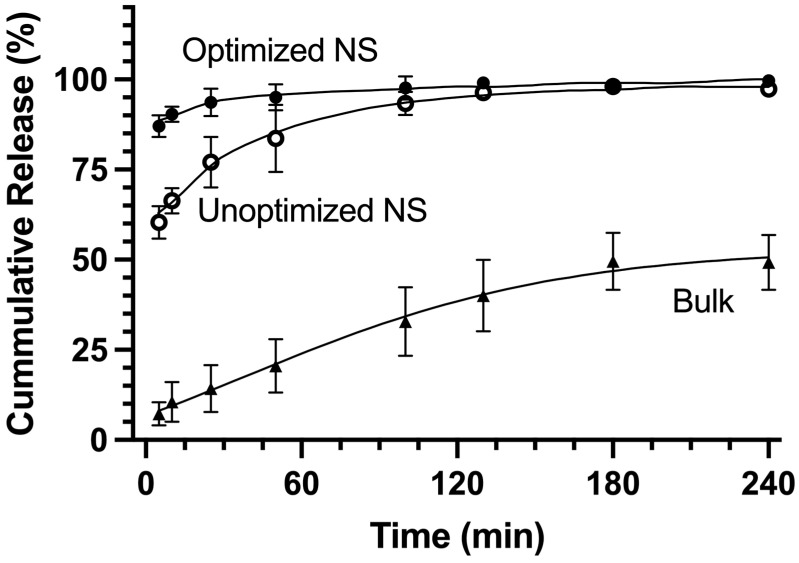
Nanosuspension (NS) optimization enhanced dissolution compared to the unoptimized NS and bulk powder. NDH-4338 bulk powder and freeze-dried optimized and unoptimized NDH-4338 NSs were added to 100 mL of 50% MeCN/50% water that was preheated to and kept at 32.5 ℃ in an incubated orbital shaker set at 200 RPM. Receptor fluid was withdrawn at each time point and centrifuged, and the resulting supernatant was analyzed by HPLC to quantify NDH-4338. The data presented were fit with a Weibull 4 model, revealing that the optimized NS shows decreased α values and increased Fmax values relative to the unoptimized NS and bulk powder, indicating faster dissolution in the optimized NS. Each point represents the mean ± SD of NDH-4338 released (*n* = 3).

Within the first 5 min, the optimized NS demonstrated approximately 87% cumulative release, compared to 60% for the unoptimized NS and 7% for the bulk powder (Figure 7). The 27% increase in burst release of the optimized NS compared to the unoptimized NS may be attributable to the 3- to 4-fold decrease in particle size. Significant differences in burst release between the NS formulations and the bulk powder are detailed in Table 3. In the optimized NS, the time to achieve 75% drug release (T_75_) was 1.8 min, considerably shorter than the 20.8 min required in the unoptimized NS, indicating a significantly faster dissolution. At the final 240 min time point, dissolution for the optimized NS reached 100% completion, while the unoptimized NS and bulk powder achieved cumulative releases of 97% and 49%, respectively (Table 3). Burst and total release from both NS formulations were significantly different from the bulk powder, highlighting the role of NS drug delivery in enhancing dissolution rates through reduced particle size.

### 3.7. Effects of NDH-4338 on NM-Induced Edema

**Figure 8:**
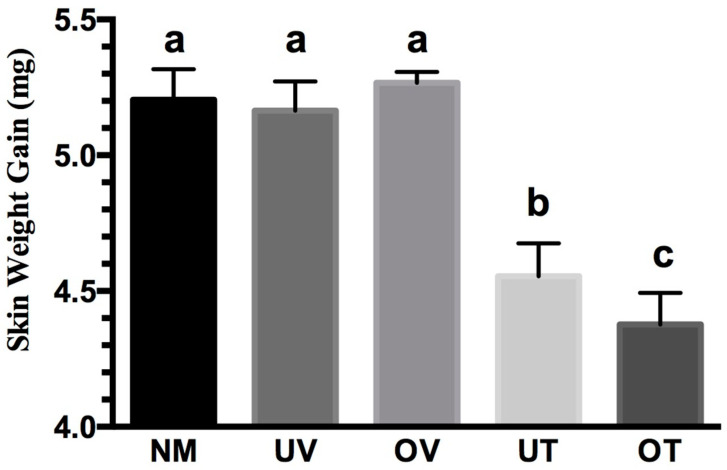
Enhanced edema mitigation in mice by optimized nanosuspension (NS) treatment (OT) compared to unoptimized NS treatment (UT). The 2% (*w*/*v*) NDH-4338 optimized nanosuspension treatment (OT) significantly mitigated skin burns in mice exposed to mechlorethamine hydrochloride (NM), in contrast to the unoptimized 2% (*w*/*v*) NDH-4338 nanosuspension treatment (UT). Clipped and depilated female CD-1 mice were exposed to 5 μmol of NM. The OT and UT groups each received 0.4 mg of NDH-4338, while the UV (Unoptimized Vehicle) and OV (Optimized Vehicle) groups were given 20 µL of the respective vehicles, with all treatments starting 1 h after NM exposure and repeated twice over 24 h. The untreated vesicant control group received NM only (NM), and naïve control mice received no vesicant or treatment. Punch biopsy weights of the NM-treated area, offset by those from the untreated naive control group, were used to assess skin edema. No significant difference was observed after the 24 h treatment course between the UV, OV, and NM control groups. The OT and UT groups yielded a significantly lower skin weight gain than the OV, UV, and NM groups, indicating a positive therapeutic effect for each formulation attributable to the drug and not the vehicle. The OT and UT groups were also significantly different, demonstrating the impact of the DOE process in reducing particle size to enhance vivo efficacy. Columns represent the natural logarithm (ln) of the group’s mean punch biopsy weight (mg) values ± SD, *n* = 5 for OV, *n* = 7 for all other groups. There were 2 experimental groups, 4 control groups, and 40 mice in total. The presented data combine results from two independent studies: *n* = 5 in one and *n* = 2 in the other. Groups not sharing the same letter designation are statistically different, as determined by one-way ANOVA with Tukey’s multiple comparison test. A *p*-value threshold below 0.05 was considered significant.

At 24 h after NM administration, the OT group showed a significant reduction in skin weight gain compared to the NM group (4.4 ± 0.1 mg versus 5.2 ± 0.1 mg) (Figure 8). A statistically significant edema reduction effect was also observed in the UT group compared to the NM control group (4.6 ± 0.1 mg versus 5.2 ± 0.1 mg). The OT and UT groups were statically different (4.4 ± 0.1 mg versus 4.6 ± 0.1 mg), highlighting the enhanced in vivo efficacy resulting from the DOE process. The NM, UV, and OV control groups showed no significant differences (5.2 ± 0.1 mg versus 5.2 ± 0.1 mg versus 5.3 ± 0.0 mg). The anti-inflammatory effect observed in the OT and UT groups were therefore attributable to NDH-4338 and not the respective vehicle (4.4 ± 0.1 mg versus 5.3 ± 0.0 mg, 4.6 ± 0.1 mg versus 5.2 ± 0.1 mg, respectively) (Figure 8). Vitamin E TPGS alone did not heal the NM burns in the UV and OV groups at two concentrations (10 mg/mL and 7.14 mg/mL, respectively).

Reported here for the first time, NDH-4338 was formulated into an NS, formulated above a 1% dose concentration, and used to effectively treat NM skin burns after only two doses administered in 24 h. The aqueous vehicle used in the optimized NS formulation may have allowed for improved dissolution and dermal penetration of the API relative to the viscous lanolin/PEG400 vehicle previously tested by our team [28,33,34]. This optimized NS formulation did not require a viscous vehicle to increase residence time on the skin to achieve a therapeutic effect. It relied on water and Vitamin E TPGS to deliver the drug. The observed burn healing efficacy in this study may be attributed to the role of Vitamin E TPGS, which has been known to enhance the permeation of poorly water-soluble compounds through the skin by improving their solubility and modifying the skin’s structure [63]. Due to the significant in vivo efficacy using the optimized NS, no additional excipients were added to the finalized formulation. While the optimized NS contained a simple Vitamin E TPGS stabilizer vehicle, it resulted in comparable NM-induced edema reduction to curcumin solid lipid nanoparticle (CSLN) hydrogels used on a similar NM skin burn model [31]. Sandhu et al. applied CSLN hydrogel treatment containing 50.6 µg of curcumin, BID, following NM exposure. They obtained a similar % decrease in NM-induced edema as that of the optimized NS used in our study. The comparable reduction in edema highlights the NM burn healing efficacy of the optimized NS, as the present study uses a 24 h study endpoint, and the CSLN hydrogel study used a 72 h endpoint. The optimized NS may have obtained a similar NM burn healing efficacy to the CSLN hydrogel formulation in a shorter period due to the approximate 6-fold reduction in particle size. Apart from the previously mentioned studies, to our knowledge, the only other publication that employs skin weight as a proxy for NM-induced edema uses a different outcome measure than our study. Tumu et al. used “fold change in wet weight”, while our depilatory double-disc NM model uses skin weight gain [64]. We felt unable to make comparisons to this study due to the different model endpoints, types of mouse skin, and outcome measures.

## 4. Conclusions

The current study represents a novel approach for reducing the particle size of a lipophilic prodrug NS, with an extensive evaluation of the optimized NS compared to the starting formulation to determine the impact of the DOE. The optimized formulation’s predicted and experimentally derived NS sizes were confirmed to be statistically equivalent within a 95% CI. The DOE process resulted in a 3- to 4-fold reduction in particle size and a more crystalline product with an increased physical stability and dissolution rate. Reported here for the first time, NDH-4338 was formulated into an NS, formulated above a 1% dose concentration, and used to effectively treat NM skin burns after only two doses administered in 24 h. These improvements in NS properties enhanced NM burn healing efficacy, underscoring the potential of this approach in revolutionizing drug formulation and delivery of poorly soluble compounds.

## Figures and Tables

**Table 1:** A Box–Behnken design was used to determine the composition of fifteen nanosuspension formulations.

Formulation	Pattern	A/S	Dose Concentration	D/S
1	000	7	1.5	3
2	−−0	5	1	3
3	+0+	9	1.5	2
4	++0	9	2	3
5	+−0	9	1	3
6	0−+	7	1	2
7	0+−	7	2	4
8	000	7	1.5	3
9	0++	7	2	2
10	+0−	9	1.5	4
11	000	7	1.5	3
12	−+0	5	2	3
13	0−−	7	1	4
14	−0−	5	1.5	4
15	−0+	5	1.5	2

**Table 2:** Weibull 4 as the preferred model for dissolution analysis of NDH-4338 in nanosuspension and bulk powder formulations. The Akaike Information Criterion (AIC), Model Selection Criteria (MSC), and R-Squared were used to assess ten mathematical models for NDH-4338 dissolution in various forms. The models included Higuchi, Higuchi F0, Zero Order, First Order, Korsmeyer–Peppas, and Weibull variants 1–4. The top three models—Higuchi F0, Korsmeyer–Peppas, and Weibull 4—were chosen based on their superior AIC, MSC, and R-Squared values. Weibull 4 was the optimal model, with the lowest AIC values and the highest R-squared and MSC values.

Model	Goodness of Fit	Bulk	Unoptimized	Optimized
Higuchi F0	R-squared	0.972 ± 0.010	0.814 ± 0.146	0.863 ± 0.068
	AIC	−37.41 ± 2.15	−45.92 ± 8.90	−27.35 ± 2.62
	MSC	3.099 ± 0.339	1.513 ± 1.132	1.579 ± 0.554
Korsmeyer–Peppas	R-squared	0.975 ± 0.005	0.898 ± 0.039	0.938 ± 0.023
	AIC	−38.31 ± 1.57	−48.40 ± 2.12	−33.38 ± 1.38
	MSC	3.211 ± 0.204	1.823 ± 0.351	2.332 ± 0.399
Weibull 4	R-squared	0.989 ± 0.007	0.973 ± 0.019	0.984 ± 0.011
	AIC	−42.02 ± 4.86	−40.72 ± 6.62	−57.04 ± 12.16
	MSC	3.676 ± 0.694	2.903 ± 1.093	3.251 ± 0.632

**Table 3:** Analysis of release data at 5 min as burst release, 240 min as total release, and T_75_ as sustained release.

	Bulk	Unoptimized NS	Optimized NS
Burst release (% released)	7.3 ± 3.3 ^a^	59.9 ± 4.5 ^b^	87.2 ± 3.2 ^c^
Total release (% released)	49.3 ± 7.6 ^a^	97.4 ± 7.0 ^b^	99.6 ± 2.0 ^b^
T_75_ (min)	NA	20.8 ± 10.5 ^a^	1.8 ± 2.4 ^b^

T_75_, representing the time required for the release of 75% of the formulation, was derived using the Weibull 4 model. Numbers in each row with different superscript letters represent significant differences (*p* < 0.05, ANOVA with Tukey’s multiple comparison tests, or two-tailed *t*-test). The data are presented as the mean percentage or time ± SD.

## Data Availability

The data presented in this study are available on request from the corresponding author.

## Supplementary Materials

The following supporting information can be downloaded at: https://www.mdpi.com/article/10.3390/pharmaceutics16040471/s1.
