# Supplementary material for: Optimizing Nanosuspension Drug Release and Wound Healing Using a Design of Experiments Approach: Improving the Drug Delivery Potential of NDH-4338 for Treating Chemical Burns
Source: Pharmaceutics. 2024 Mar 27;16(4):471. doi: 10.3390/pharmaceutics16040471 (PMC11053863; doi:10.3390/pharmaceutics16040471)
Supplement: Supplementary file 1 [file pharmaceutics-16-00471-s001.zip › Supplementary Materials Word Document version (with captions).pdf]

Supplementary Materials:

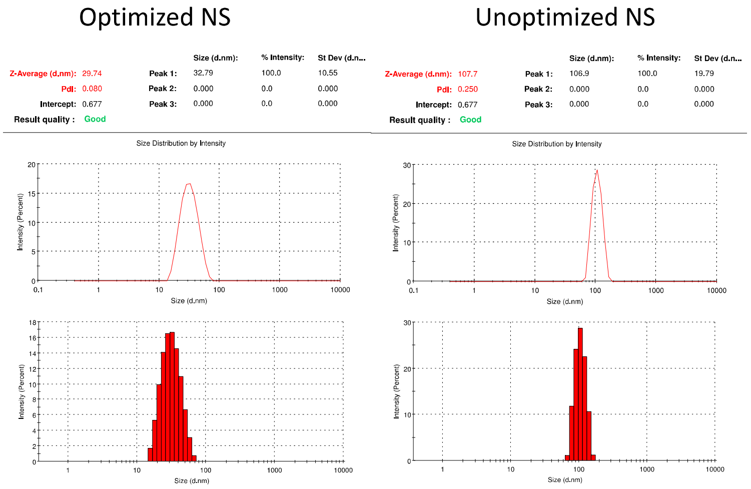

Figure S1. Dynamic Light scattering intensity plots of the optimized and unoptimized nanosuspensions (NSs).

Table S1. The effect summary of the model shows that the removal of AS*DC improved the p-values of all other effects. Logworth is a statistical measure used in bioinformatics that represents the negative base 10 logarithm of the p-value, providing a scale for assessing the statistical significance of an observation, where higher values indicate greater significance. The three factors are antisolvent/solvent ratio (A/S), dose concentration (DC), and drug/stabilizer ratio (D/S). An examination of the original effect summary (left) reveals that the AS*DC term's p-value stands at 0.45193, nearly twice as high as other effects. In the final effect summary (right), the removal of AS*DC lowered the p-value of all other effects. Removal of other effects increased the p-value of all other effects and were thus retained in the model.

	Original		After removal	
**Source**	**LogWorth**	**P Value**	**LogWorth**	**P Value**
DC (1,2)	3.016	0.00096	3.416	0.00038
D/S (2,4)	1.529	0.02960	1.677	0.02105
A/S (5,9)	1.197	0.06354	1.300	0.05016
AS*DS	0.890	0.12873	0.957	0.11052
DC*DS	0.808	0.15546	0.866	0.13616
DS*DS	0.791	0.16198	0.846	0.14246
DC*DC	0.644	0.22704	0.686	0.20628
AS*AS	0.603	0.24924	0.641	0.22834
AS*DC	0.345	0.45193		

Table S2. Stability regression analysis for size and polydispersity index (PDI) of Unoptimized and Optimized nanosuspensions.

	Unoptimized NS Size	Optimized NS Size		Unoptimized NS PDI	Optimized NS PDI
**Best-fit values**			**Best-fit values**		
**Slope**	-0.7211	0.01504	**Slope**	0.004103	0.0003472
**Y-intercept**	132.4	17.22	**Y-intercept**	0.2505	0.1882
**X-intercept**	183.6	-1145	**X-intercept**	-61.05	-541.9
**1/slope**	-1.387	66.48	**1/slope**	243.7	2880
**Std. Error**			**Std. Error**		
**Slope**	0.1207	0.03037	**Slope**	0.0006929	0.0002036
**Y-intercept**	7.387	1.858	**Y-intercept**	0.0424	0.01246
**95% Confidence Intervals**			**95% Confidence Intervals**		
**Slope**	-0.9901 to -0.4521	-0.05262 to 0.08270	**Slope**	0.002559 to 0.005647	-0.0001065 to 0.0008010
**Y-intercept**	116.0 to 148.9	13.08 to 21.36	**Y-intercept**	0.1560 to 0.3449	0.1604 to 0.2159
**X-intercept**	144.8 to 266.3	-infinity to -166.2	**X-intercept**	-129.3 to -28.81	-infinity to -206.1
**Goodness of Fit**			**Goodness of Fit**		
**R squared**	0.7811	0.02395	**R squared**	0.7781	0.2252
**Sy.x**	15.87	3.992	**Sy.x**	0.09108	0.02677
**Is slope significantly non-zero?**			**Is slope significantly non-zero?**		
**F**	35.68	0.2454	**F**	35.06	2.907
**DFn, DFd**	1, 10	1, 10	**DFn, DFd**	1, 10	1, 10
**P value**	0.0001	0.631	**P value**	0.0001	0.119
**Deviation from zero?**	Significant	Not Significant	**Deviation from zero?**	Significant	Not Significant
**Equation**	Y = -0.7211*X + 132.4	Y = 0.01504*X + 17.22	**Equation**	Y = 0.004103*X + 0.2505	Y = 0.0003472*X + 0.1882
**Data**			**Data**		
**Number of X values**	12	12	**Number of X values**	12	12
**Maximum number of Y replicates**	1	1	**Maximum number of Y replicates**	1	1
**Total number of values**	12	12	**Total number of values**	12	12
**Number of missing values**	0	0	**Number of missing values**	0	0

Table S3. Additional parameters for the three models with the best fit. Weibull 4 model's parameters include *α* (scale), β (shape), Ti (lag time), and Fmax (maximum fraction of drug release at infinite time). Higuchi F0's key variable is the Higuchi release rate constant (kH), while Korsmeyer–Peppas includes the release rate constant (kKP) and diffusion exponents (n).

		**Bulk**	**Optimized**	**Unoptimized**
Higuchi F0	kH	0.035	0.009	0.029
Korsmeyer–Peppas	kKP	0.031	0.835	0.503
	n	0.563	0.0334	0.129
Weibull 4	α	30354.929	15.924	610.610
	β	1.792	0.462	1.111
	Ti	–48.808	–24.894	–49.704
	Fmax	0.541	1.639	0.990
